# UNDERSTANDING THE EXPERIENCE OF EUSTRESS AMONG OLDER ADULTS WITH CHRONIC CONDITIONS

**DOI:** 10.1093/geroni/igac059.1882

**Published:** 2022-12-20

**Authors:** Jaesung An, Laura Payne, Toni Liechty, Yoshitaka Iwasaki

**Affiliations:** California State University East Bay, Cupertino, California, United States; University of Illinois Urbana-Champaign, Champaign, Illinois, United States; University of Illinois Urbana-Champaign, Champaign, Illinois, United States; San Jose State University, San Jose, California, United States

## Abstract

In their longitudinal study of stress and health among adults, McGonigal and colleagues (2013; 2016) suggested that how person perceives stress is a key factor affecting their actual physiological health. This is related to the concept of eustress (i.e., positive response to a given stressor) which has received very little attention in the literature. Besides, older adults with chronic conditions deal with symptoms that are difficult to manage and can contribute to chronic stress, which can then affect their health status and quality of life. Thus, helping older adults to shift their perceptions and interpretations of stressors from distress to eustress, rather than trying to avoid stress seems to be a reasonable approach to help them experience healthy aging. The purpose of this study was to explore the experience of eustress among older adults with chronic conditions in the context of leisure. Sixteen participants were recruited from three settings in a Midwestern city: a retirement community, an assisted living center, and a community senior program. Findings showed that participants’ health conditions were one of their greatest stressors, however, they modified their leisure engagement based on their conditions to enable their engagement in valued leisure activities. In the context of leisure, five themes emerged as facilitators that helped older adults to experience eustress: leisure satisfaction, sense of control, high levels of perseverance, positive attitude, and spiritual belief. Direct quotes and further discussion on how the themes are represented in the stress, leisure, and gerontology literature will be presented.

